# Giant paraumbilical hernia causing gastric outlet obstruction in a young man with class III obesity: a case report and literature review

**DOI:** 10.3389/fsurg.2026.1873927

**Published:** 2026-06-24

**Authors:** Guo Hou Loo, Meng Xian Chan, Zhun Shen Tan, Nik Ritza Kosai

**Affiliations:** 1 Upper Gastrointestinal and Metabolic Surgery Unit, Department of Surgery, Faculty of Medicine, Universiti Kebangsaan Malaysia, Hospital Canselor Tuanku Muhriz, Kuala Lumpur, Malaysia; 2 Faculty of Medicine, Universiti Kebangsaan Malaysia, Hospital Canselor Tuanku Muhriz, Kuala Lumpur, Malaysia

**Keywords:** bioelectrical impedance, case report, class III obesity, gastric outlet obstruction, loss of domain, paraumbilical hernia, rives-stoppa repair, ventral hernia

## Abstract

**Background:**

Gastric outlet obstruction (GOO) caused by a paraumbilical hernia is exceptionally rare. The stomach is normally tethered by its peritoneal attachments and seldom migrates into a midline ventral defect; reported cases have almost exclusively involved elderly patients, in whom acquired ligamentous laxity has been invoked as a permissive factor.

**Case description:**

A 38-year-old man with lifelong class III obesity (peak BMI 48.2 kg/m^2^), severe obstructive sleep apnoea and a sliding hiatal hernia presented with a one-year history of daily post-prandial non-bilious vomiting, learned self-induced emesis to relieve hernia distension, profound unintentional weight loss (from 126.4 to 89 kg, a 30% reduction) and chronic constipation. Examination revealed a giant irreducible paraumbilical hernia. Contrast-enhanced computed tomography (CT) demonstrated a fascial defect measuring 5.6 × 7.5 cm (transverse × cranio-caudal) containing small bowel, transverse colon, omentum and stomach, with a transition point at the distal antrum and pylorus indicating extrinsic GOO and no evidence of malignancy. Endoscopy showed two-level extrinsic compression at the gastric body and pylorus, retained food in the proximal stomach, and could not easily be passed across the pyloric constriction; a nasojejunal feeding tube was placed under direct visualisation. Pre-operative biochemistry was unremarkable apart from hypokalaemia (potassium 2.78 mmol/L), which was corrected before surgery; serial bioelectrical impedance and hand-grip dynamometry suggested preserved muscle mass during prehabilitation. An open Rives–Stoppa retrorectus mesh repair was performed; the intra-operative defect measured 5 × 10 cm (transverse × cranio-caudal) and the contents were viable. Recovery was uneventful. At the most recent review, he was asymptomatic with a full oral diet and regular bowel function.

**Conclusion:**

GOO from a stomach-containing paraumbilical hernia can occur in young patients with severe obesity, where chronically raised intra-abdominal pressure and progressive intra-sac extrusion of viscera through a stable but capacious defect, rather than ligamentous laxity, account for migration of the stomach into the sac. A high index of suspicion, multi-detector CT, formal volumetric loss-of-domain assessment, multidisciplinary optimisation, and retromuscular (Rives-Stoppa) repair offered satisfactory short-term resolution in this patient, with longer follow-up required to confirm durability. The case is reported in accordance with the CARE 2013 reporting guidelines.

## Introduction

Paraumbilical hernias are acquired defects of the linea alba immediately adjacent to the umbilicus and account for a substantial proportion of adult abdominal-wall hernias. In adults they are predominantly driven by sustained elevation of intra-abdominal pressure, with obesity, multiparity, ascites, chronic cough, heavy lifting and previous abdominal surgery as the principal risk factors (1, 2). Although most remain asymptomatic or cause only intermittent local discomfort, complications of incarceration, strangulation and bowel obstruction are well recognised (1, 3).

Gastric outlet obstruction (GOO) is itself an uncommon clinical syndrome. In contemporary practice, malignant disease particularly gastric and pancreatic adenocarcinoma, is the most frequent cause in adults, followed by benign processes such as peptic ulcer disease, gastric polyps, ingested caustics, gastric bezoars and Bouveret syndrome (4–6). Mechanical extrinsic compression of the gastric outlet by an abdominal-wall hernia is a far less commonly recognised aetiology and is not routinely listed in standard differential diagnoses (4, 5).

GOO produced by a stomach-containing paraumbilical hernia is exceptional. The stomach is normally anchored by its gastrocolic, gastrohepatic, gastrosplenic and phrenicogastric ligaments and is a relatively immobile viscus; reports of gastric migration into a ventral defect have therefore largely been confined to elderly patients, in whom acquired ligamentous laxity is presumed to permit such displacement (3, 7, 8). Even in the closely related context of stomach-containing groin hernias, fewer than 100 cases have been reported in two centuries of surgical literature, with GOO complicating only a minority (3). Cases of paraumbilical, supra-umbilical or umbilical port-site hernias presenting with GOO have been described almost exclusively in older women (7, 9), and reports involving young patients are very limited.

We describe a 38-year-old man with class III obesity, severe obstructive sleep apnoea and a previously deferred bariatric pathway who presented with a one-year history of post-prandial vomiting, self-induced emesis and dramatic unintentional weight loss caused by extrinsic GOO from a giant paraumbilical hernia containing the stomach, transverse colon, small bowel and omentum, and which was managed successfully by an open Rives-Stoppa retrorectus mesh repair. We review the relevant literature and discuss the clinical, imaging and operative considerations that distinguish this presentation. The case is reported in accordance with the CARE 2013 reporting guidelines (10).

For the literature review component, we conducted a focused narrative review of the English-language literature relevant to this case, including stomach-containing ventral hernias and gastric outlet obstruction secondary to ventral hernia, complex ventral hernia repair, and the technical adjuncts used here, namely bioelectrical impedance analysis and hand-grip dynamometry for nutritional and functional assessment, CT-based volumetric assessment of loss of domain (Tanaka and Sabbagh methods), and intravesical manometry for intra-abdominal pressure measurement. PubMed, Embase and the Cochrane Library were searched from inception to May 2026 using combinations of the terms ‘paraumbilical hernia’, ‘umbilical hernia’, ‘ventral hernia’, ‘gastric outlet obstruction’, ‘loss of domain’, ‘Rives-Stoppa repair’, ‘bioelectrical impedance’, ’sarcopenia’, ‘intra-abdominal hypertension’ and ‘bariatric surgery’. Articles were screened by title and abstract, with full-text review of those judged relevant; reference lists of included papers were hand-searched for additional citations. The review is narrative rather than systematic and is intended to contextualise the present case rather than to provide an exhaustive synthesis.

## Case presentation

A 38-year-old male government computer technician was referred to our upper gastrointestinal and metabolic surgery clinic for evaluation of a long-standing paraumbilical hernia and ongoing obesity management. He was an ex-smoker with no previous abdominal surgery.

His background was notable for lifelong class III obesity (heaviest documented weight 151 kg, peak BMI 48.2 kg/m^2^; weight at first surgical contact 124–131 kg, BMI 39.6–41.8 kg/m^2^), severe obstructive sleep apnoea (initial apnoea–hypopnoea index 96.3; lowest oxygen saturation 53%) on continuous positive airway pressure (CPAP) therapy under a tertiary respiratory service, and a sliding hiatal hernia with reflux oesophagitis (Los Angeles grade A–B) maintained on a proton-pump inhibitor. Bariatric surgery had previously been worked up but was deferred for several years owing to active tobacco use and poor CPAP adherence. The full weight, BMI and biochemical trajectory is summarised in Table 1. Of note, the baseline contrast-enhanced CT in November 2022 demonstrated a right paraumbilical hernia containing small bowel, transverse colon, the distal antrum/pylorus, mesenteric fat and SMA branches, with no proximal gastric dilatation and no radiological or clinical features of obstruction (Table 1, Figures 2A,C); the patient was asymptomatic at that time and the hernia was clinically reducible at outpatient review. In the absence of incarceration, obstruction or symptomatic distress, expectant management with prioritisation of weight optimisation and a bariatric pathway was elected rather than early elective repair, particularly in view of his active tobacco use and poor CPAP adherence, both of which substantially increased his peri-operative risk. Throughout this period he remained under regular outpatient follow-up by our upper-gastrointestinal and metabolic surgery service, with serial clinical review and continued respiratory and lifestyle optimisation; the initial plan was for combined metabolic bariatric surgery and ventral hernia repair once tobacco use and CPAP adherence had been optimised, but the patient was not keen on the bariatric component and accordingly chose to defer surgical intervention while he remained asymptomatic. The decision to postpone operative repair therefore reflected shared decision-making within an active follow-up pathway rather than loss to care. Definitive repair became indicated only when sustained extrinsic GOO and profound unintentional weight loss developed during the year preceding admission.

**Table 1:** Clinical timeline (CARE-compliant).

Date/time-point	Event	Clinical detail
Years prior	Onset and gradual enlargement of paraumbilical hernia	Sedentary occupation; class III obesity (peak 151 kg, peak BMI 48.2 kg/m^2^)
∼3–5 years prior	Bariatric work-up	Deferred due to active tobacco use and suboptimal CPAP compliance for severe OSA (AHI 96.3; nadir SpO₂ 53%)
8 Nov 2022	Baseline CECT abdomen/pelvis	Right paraumbilical hernia containing small bowel, transverse colon, distal antrum/pylorus, mesenteric fat and SMA branches; hernial neck 5.7 × 7.5 cm (W × CC); no proximal gastric dilatation; no GOO
∼12 months before admission	Symptom onset	Daily post-prandial non-bilious vomiting; learned self-induced emesis to relieve hernia distension
18 Jul 2025	Repeat CECT abdomen/pelvis	Hernia now contains stomach, D1, proximal D2, small bowel, transverse colon and omentum; hernial neck 5.6 × 7.5 cm (W × CC); no proximal gastric dilatation at this scan; no malignancy
Over preceding 12 months	Progressive unintentional weight loss	126.4 → 89 kg (≈30%)
19 Nov 2025	Pre-operative biochemistry	K⁺ 2.78 mmol/L (low); albumin 34 g/L (low); Na⁺ 139; creatinine 75 µmol/L; eGFR >90; LFTs normal
20 Nov 2025	OGDS + nasojejunal tube placement	Extrinsic compression at gastric body and at pylorus; retained antral food; small Forrest III D1 ulcer; NJ tube placed under endoscopic + fluoroscopic guidance
20 Nov 2025	BIA #1 (pre-prehabilitation)	Weight 89.5 kg; BMI 28.6; SMM 36.9 kg; FFM 66.2 kg; BMR 1799 kcal; body fat 26.1%
20 Nov – Dec 2025	Pre-operative optimisation	NJ enteral feeding; NG drainage 500–1000 mL/day for 2–3 days then NG discontinued; K⁺ replacement; CPAP optimisation; VTE prophylaxis; physiotherapy and incentive spirometry
28 Nov 2025	BIA #2 (mid-prehabilitation)	Weight 89.6 kg; BMI 28.6; SMM 37.7 kg; FFM 67.2 kg
10 Dec 2025	BIA #3 + hand-grip dynamometry	Weight 93.3 kg; BMI 29.8; SMM 38.9 kg; FFM 69.6 kg; BMR 1873 kcal; grip 52 kg dominant/51 kg non-dominant (above age- and sex-matched reference)
Day 0 (index surgery)	Open Rives–Stoppa retrorectus mesh repair	Adhesiolysis after GA + neuromuscular blockade; defect 5 × 10 cm (W × CC); contents viable; Ventralight ST 10 × 15 cm in retrorectus plane with ≥3 cm overlap; linea alba closed with looped polyamide 1
Day 1	Extubation	After 24 h elective ICU ventilation; saturating room air
Day 3	Oral diet resumed	Tolerating diet; no further emesis
Day 6	Discharge	Pain-controlled; ambulant
∼1 month	First outpatient review	Asymptomatic; weight 90 kg (BMI 28.7)
4 May 2026 (∼5 months)	Most recent review	Asymptomatic; full oral diet; regular bowel function; weight 118 kg (BMI 37.7, class II); re-referred for bariatric assessment

AHI, apnoea–hypopnoea index; BIA, bioelectrical impedance analysis; BMI, body mass index; BMR, basal metabolic rate; CC, cranio-caudal; CECT, contrast-enhanced computed tomography; CPAP, continuous positive airway pressure; FFM, fat-free mass; GA, general anaesthesia; GOO, gastric outlet obstruction; ICU, intensive care unit; NG, nasogastric; NJ, nasojejunal; OGDS, oesophago-gastro-duodenoscopy; OSA, obstructive sleep apnoea; SMM, skeletal muscle mass; VTE, venous thromboembolism; W, transverse width.

He was admitted for symptoms compatible with GOO attributed to a giant, irreducible paraumbilical hernia of approximately one year's duration, on a background of a hernia that had been gradually enlarging over a much longer period. Symptoms had evolved insidiously and consisted of daily post-prandial non-bilious vomiting, typically at midday and at night, precipitated by moderate volumes of food or fluid. He had learned to induce vomiting himself in order to relieve a sensation of fullness and visible enlargement of the hernia after meals. Repeated vomiting and food avoidance led to marked unintentional weight loss, from 126.4 kg to 89 kg over one year (a 30% reduction in body weight; BMI 28.6 kg/m^2^ at admission), accompanied by subjective anorexia. He also reported chronic constipation, with bowel motions every four to five days, hard stools requiring straining, intermittent reliance on lactulose, and a sensation of incomplete evacuation.

On examination, he was alert, comfortable, haemodynamically stable, and saturating on room air. The abdomen was soft and non-tender. A giant, irreducible paraumbilical hernia dominated the clinical picture. There were multiple superficial scratch and abrasion wounds over the dependent overlying skin, but no erythema, ulceration, discharge or signs of strangulation. The hernia was non-tender, and there were no peritoneal signs (Figure 1).

**Figure 1:**
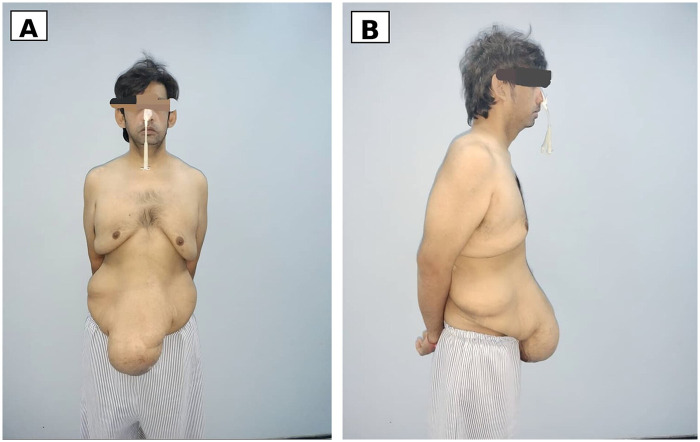
Pre-operative clinical photographs of the giant paraumbilical hernia. **(A)** Anterior view showing a pendulous paraumbilical hernia (clinical skin envelope approximately 30 × 30 cm) extending from the supra-umbilical region to mid-thigh, together with bilateral pseudo-gynaecomastia and redundant abdominal soft tissue consistent with class III obesity at peak weight and substantial unintentional weight loss. **(B)** Lateral view demonstrating the marked anterior projection of the hernia sac. The naso-jejunal feeding tube was *in situ* for simultaneous gastric decompression and pre-operative enteral nutritional support. The patient's body weight at this point was 89 kg, having decreased from a baseline of 126.4 kg over one year owing to extrinsic gastric outlet obstruction and learned self-induced emesis to relieve hernia distension.

Upper gastrointestinal endoscopy was performed with a slim-caliber transnasal endoscope (approximately 5 mm outer diameter) under continuous direct visualisation, both to exclude an intrinsic malignant or peptic cause of GOO and to enable direct-vision placement of a nasojejunal feeding tube for pre-operative enteral nutrition. This demonstrated reflux oesophagitis (Los Angeles grade A) and a grade 2 sliding hiatal hernia. The proximal stomach contained retained, semi-digested food. Two distinct points of extrinsic luminal compression were identified along the stomach: the first at the body, through which the endoscope was carefully advanced under direct vision without force, and a second at the pylorus, where the antrum was pooled with food and where the scope could not be readily traversed and was not forced. A small (Forrest III) duodenal-bulb (D1) ulcer was noted once D1 was reached. There was no pyloric mass, peptic stenosis or mucosal lesion of a degree sufficient to explain the GOO. The pattern of two-level extrinsic compression along an otherwise normal mucosa was consistent with mechanical angulation of the stomach as it traversed the hernial neck. Mucosal biopsies and Helicobacter pylori testing were not performed at the index endoscopy: the principal aims were to exclude an intrinsic malignant or stricturing cause of the obstruction and to enable safe direct-vision placement of the nasojejunal feeding tube, and the macroscopic mucosal appearance apart from the small Forrest III duodenal-bulb ulcer did not raise additional concern. The patient was maintained on proton-pump inhibitor therapy, with the intention of formal H. pylori assessment at planned post-operative surveillance endoscopy; we acknowledge that earlier sampling at the index endoscopy would have been preferable and would now be undertaken routinely in patients with comparable findings.

Because the pylorus could not be readily negotiated, a nasojejunal (NJ) feeding tube was inserted under direct endoscopic visualisation using a guidewire technique, with the tip confirmed by fluoroscopy in the proximal jejunum, to provide simultaneous gastric decompression and enteral nutritional support.

Colonoscopy was technically abandoned because of severe looping of the colon within the hernia sac and an acute angulation produced by the defect, itself an unusual finding that supported the mechanical role of the hernia.

Multi-detector contrast-enhanced computed tomography (CT) of the abdomen and pelvis is the preferred investigation in any patient with a giant ventral hernia, particularly when atypical upper-gastrointestinal symptoms are present (1). In our patient, CT demonstrated a paraumbilical hernia containing small bowel, the duodenal bulb (D1), proximal D2, transverse colon, omentum and stomach (Figures 2B,D). The hernial neck measured 5.6 × 7.5 cm (transverse ×  cranio-caudal). The stomach was markedly distended, fluid- and air-filled, and occupied the central and right paraumbilical region; the mechanical transition point was at the level of the distal antrum and pylorus, where the gastric outlet was extrinsically angulated as it crossed the fascial defect. The small bowel and colon distal to this transition were decompressed, in keeping with mechanical obstruction at the gastric outlet rather than at a more distal level. There was no gastric wall thickening, mass lesion or peri-gastric fat stranding to suggest an alternative malignant or inflammatory cause.

**Figure 2:**
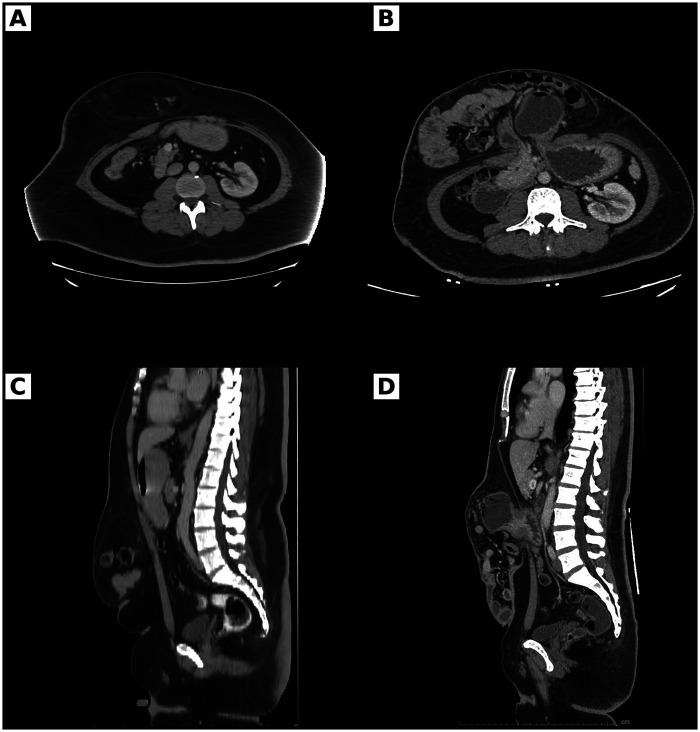
Evolution of the paraumbilical hernia and its contents on cross-sectional imaging between 2022 and 2025. **(A)** Axial contrast-enhanced CT, November 2022. **(B)** Axial contrast-enhanced CT, July 2025. **(C)** Sagittal contrast-enhanced CT, November 2022. **(D)** Sagittal contrast-enhanced CT, July 2025. On the 2022 imaging (panels A and C), the hernial sac contained small bowel, transverse colon, the distal antrum/pylorus, mesenteric fat and SMA branches, with no proximal gastric dilatation and no radiological evidence of obstruction; the hernial neck measured 5.7 × 7.5 cm (transverse × cranio-caudal). On the 2025 imaging (panels B and D), the hernial neck dimensions were essentially unchanged (5.6 × 7.5 cm), but the sac now accommodated substantially more viscera: a markedly distended, fluid- and air-filled stomach occupied the central and right paraumbilical region, with the mechanical transition point at the level of the distal antrum and pylorus where the gastric outlet is extrinsically angulated as it crosses the fascial defect. The small bowel and colon distal to this transition are decompressed, in keeping with mechanical obstruction at the gastric outlet rather than at a more distal level. There is no gastric wall thickening, mass lesion or peri-gastric fat stranding to suggest an alternative malignant or inflammatory cause.

Formal volumetric assessment of loss of domain (LOD) using the Tanaka index (hernia sac volume ÷ abdominal cavity volume; threshold >0.25) (11) or the Sabbagh method (hernia sac volume ÷ total peritoneal volume; threshold >0.20) (12) was not performed pre-operatively. A qualitative review of the cross-sectional imaging by the operating team suggested that the herniated volume was below the threshold for clinically significant LOD, a judgement that was confirmed intra-operatively. The absence of formal volumetric LOD calculation is acknowledged as a limitation.

On admission, serum biochemistry revealed hypokalaemia (potassium 2.78 mmol/L) and mild hypoalbuminaemia (albumin 34 g/L; reference 35–50). Sodium (139 mmol/L), urea (5.0 mmol/L), creatinine (75 µmol/L) and acid–base status were normal; eGFR was >90 mL/min/1.73 m^2^. Liver function tests and corrected calcium were within reference. Potassium was replaced enterally during prehabilitation, and the value was within the reference range before the day of surgery.

Given the magnitude of weight loss (30%) and the protracted period of vomiting, nutritional status was monitored objectively using serial bioelectrical impedance analysis (BIA; InBody platform) and Jamar-style hand-grip dynamometry, supplemented by serum albumin and total protein. On the day of NJ tube placement (20 November 2025), the patient weighed 89.5 kg (BMI 28.6 kg/m^2^) with skeletal muscle mass (SMM) 36.9 kg, fat-free mass 66.2 kg, body fat mass 23.3 kg (26.1%), basal metabolic rate 1799 kcal, and serum albumin 34 g/L. After approximately one week of NJ enteral feeding, SMM had increased to 37.7 kg and fat-free mass to 67.2 kg at a near-identical body weight (89.6 kg), reflecting prehabilitation-related lean mass gain rather than fluid overload. Just before surgery, weight had risen to 93.3 kg with further gain in SMM to 38.9 kg, fat-free mass 69.6 kg and basal metabolic rate 1873 kcal. Hand-grip strength was preserved throughout the pre-operative period and lay well above the age- and sex-matched reference range (right/dominant 52 kg, left/non-dominant 51 kg; reference 24–38 kg and 23–36 kg, respectively), arguing against functionally significant sarcopenia despite the recent dramatic weight loss (13). This trajectory of preserved grip strength with progressive lean-mass gain on BIA informed the timing of operative intervention.

The patient was managed by a multidisciplinary team comprising upper-gastrointestinal surgery, anaesthesia, respiratory medicine, dietetics and physiotherapy. Placement of the NJ tube was technically demanding because the gastric body and antrum were partially herniated into the sac, displacing the pyloro-duodenal axis anteriorly, and the angulated outlet could not be traversed blindly. Following placement, the indwelling nasogastric (NG) lumen drained 500–1000 mL of bilio-gastric fluid per day for the first 2 to 3 days, after which NG output reduced substantially and the NG lumen was discontinued while NJ enteral feeding was continued through to the day of surgery. CPAP therapy was optimised, mechanical and pharmacological venous thromboembolism prophylaxis was instituted, and incentive spirometry commenced. The patient was counselled regarding operative risks specific to a giant ventral hernia, including the small but real possibility of intra-abdominal hypertension, abdominal compartment syndrome, respiratory failure, surgical-site occurrence and recurrence. Written informed consent for surgery and for publication of this anonymised report was obtained.

An open Rives-Stoppa retrorectus mesh repair was performed under general anaesthesia. An elliptical incision was made incorporating the redundant overlying skin and the apex of the hernia sac. The hernia was clinically irreducible on awake examination. Following induction of general anaesthesia with full neuromuscular blockade, the sac was opened, and multiple omental and entero-omental adhesions between hernia contents and the sac, and between adjacent loops were identified and divided. Only after complete adhesiolysis were the small bowel, transverse colon, omentum and stomach delivered out of the sac, inspected, found to be entirely viable, and reduced into the abdominal cavity. Reduction was therefore neither spontaneous nor effortless; it was achieved only after pharmacological abdominal-wall relaxation combined with formal adhesiolysis. The fascial defect, measured with the abdomen relaxed, was 5 × 10 cm (transverse × cranio-caudal). The redundant sac was excised, and the peritoneum was closed with continuous polyglactin 1.

The posterior rectus sheaths were developed off the bilateral recti muscles to create a retromuscular space and were re-approximated with continuous polyglactin 1. The choice of mesh deserves comment. Although the Bard Ventralight™ ST composite mesh (10 × 15 cm) is marketed and primarily indicated for intra-peritoneal placement, the posterior rectus sheath in our patient was thin and centrally attenuated at the apex of the defect; we were concerned that the interrupted transfascial polypropylene sutures used to anchor the mesh could cheese-wire through the attenuated posterior layer and expose intra-peritoneal viscera directly to the polypropylene component of the prosthesis. The visceral-surface anti-adhesive coating of Ventralight ST was therefore retained as a safety margin against bowel adherence to mesh in the event of partial posterior-layer breakdown; a standard uncoated macroporous polypropylene mesh would otherwise have been our default in the retrorectus plane. The mesh was positioned in the retrorectus plane with at least 3 cm of fascial overlap on all sides and anchored with interrupted polypropylene 2–0 transfascial sutures. The linea alba was reapproximated with looped polyamide 1, the anterior rectus sheath was closed with continuous polyglactin 1, and a 10-Fr Radivac drain was placed above the anterior rectus sheath and exteriorised through the right iliac fossa. The subcutaneous layer was closed with polyglactin 2–0 and the skin with surgical staples.

Given the size of the hernia, the volume of reduced viscera and the patient's baseline cardiorespiratory profile, he was electively kept intubated and ventilated for 24 h in the intensive care unit to mitigate the risks of intra-abdominal hypertension, abdominal compartment syndrome and post-operative respiratory failure. Monitoring during this interval comprised continuous peak inspiratory airway pressure and dynamic lung compliance, hourly urine output, serial arterial lactate and clinical assessment of abdominal-wall tension; direct intravesical (Foley) manometry was not performed. We acknowledge that intravesical pressure measurement is the World Society of the Abdominal Compartment Syndrome (WSACS) recommended reference technique for the diagnosis of intra-abdominal hypertension (14), and its omission represents a methodological limitation of our perioperative pathway.

He was extubated the following day, saturating on room air. Enteral feeding was reintroduced according to an enhanced-recovery protocol, and he was tolerating an oral diet from Day 3 without further vomiting. The 10-Fr Radivac drain was monitored daily; the output was serosanguinous and declined progressively to less than 30 mL/24 h, and the drain was removed on Day 5, prior to discharge, with no clinical or ultrasonographic evidence of seroma, haematoma or surgical-site occurrence. He was discharged on Day 6 on oral analgesia, a proton-pump inhibitor and a short course of oral antibiotics, ambulant and pain-controlled. Because the operation incorporated excision of the redundant overlying skin in addition to placement of a permanent retrorectus prosthesis, the post-discharge oral antibiotic course was administered as prophylaxis against surgical-site infection, secondary seroma infection and mesh contamination, in keeping with our institutional protocol for this type of combined abdominal-wall reconstruction in patients with obesity. At one-month follow-up, he was asymptomatic, tolerating a full diet, with regular bowel motions, and the surgical wound was well-healed without seroma, infection or early recurrence. The most recent clinical review was on 4 May 2026, providing approximately five months of post-operative follow-up; he remained asymptomatic with normal bowel function. With resolution of the obstruction, his oral intake normalised and his weight increased substantially, from 89 kg pre-operatively to 90 kg at one month and 118 kg at most recent review (BMI 37.7 kg/m^2^, class II obesity) (Figure 3). He was re-referred for ongoing weight management with reconsideration of bariatric surgery once the abdominal wall has fully consolidated.

**Figure 3:**
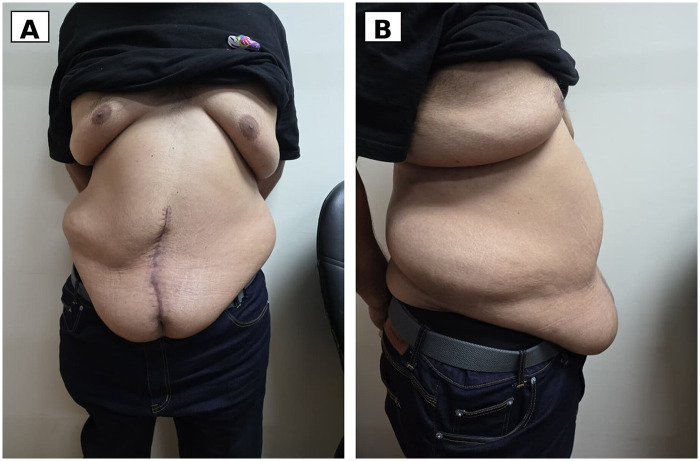
Post-operative clinical photographs at the most recent review (4 May 2026, approximately five months) following open rives–stoppa retrorectus mesh repair. **(A)** Anterior view demonstrating a well-healed midline laparotomy scar, complete reduction of the previous paraumbilical hernia, and restoration of a flat abdominal contour without clinical signs of recurrence. **(B)** Lateral view confirming absence of any pendulous hernia sac; mild residual lower-abdominal panniculus reflects the underlying obesity. The patient's body weight at this visit was 118 kg (BMI 37.7 kg/m^2^, class II obesity), an increase of 29 kg from the pre-operative weight of 89 kg, reflecting resumption of normal oral intake following relief of the obstruction and the re-emergence of his underlying obesity. He has been re-referred for bariatric surgical assessment now that wound consolidation permits.

## Discussion

GOO is a clinical syndrome of impaired transit at the distal stomach or proximal duodenum (4–6). Benign disease (most often peptic stricture and bezoars) and malignant infiltration of the pyloro-duodenal region account for most cases (4–6); extrinsic mechanical compression by an abdominal-wall hernia is recognised but distinctly unusual, and is most often described with an incarcerated stomach in a hiatal hernia or, rarely, in a ventral hernia (7–9, 15).

Stomach-containing groin hernias, although themselves uncommon, are the best-characterised extra-anatomical site of gastric herniation; a recent systematic review identified 91 cases over two centuries, with GOO complicating roughly 18% (3). Cases of stomach-containing umbilical, paraumbilical, supra-umbilical or umbilical port-site hernias presenting with GOO have been reported in only a small number of single-case publications, predominantly involving elderly female patients in whom acquired gastric ligamentous laxity has been invoked as a permissive factor (7, 8, 15). Lim and Hattori described GOO from a paraumbilical hernia in an 80-year-old woman with a long-standing supra-umbilical defect (7), and Boyapati and colleagues described pyloric incarceration within an umbilical port-site hernia in an 81-year-old woman (8).

### Distinguishing features of the present case

Three features distinguish the present case from previously reported episodes. First, the patient was young (38 years), well below the typical age range for stomach-containing ventral hernias and male, contrasting with the female and geriatric demographic predominance noted in the existing literature (3, 7, 8). Second, the dominant predisposing factor was not ligamentous laxity but chronically raised intra-abdominal pressure associated with lifelong class III obesity (peak weight 151 kg, peak BMI 48.2 kg/m^2^), combined with progressive intra-sac extrusion of additional viscera through a stable but capacious paraumbilical fascial defect over many years (1, 16, 17). Sustained intra-abdominal hypertension is a recognised driver of primary and recurrent ventral hernias and is associated with larger defects, higher recurrence rates and increased peri-operative complications across higher BMI classes (16, 17, 22). Over time, sustained intra-abdominal pressure forced progressively more viscera through the same defect: small bowel, transverse colon and the distal antrum/pylorus were already herniated on the baseline 2022 CT, and proximal migration of the gastric body, duodenal bulb and proximal D2 was documented on the 2025 CT (Figure 2); notably, the hernial neck dimensions themselves remained essentially unchanged across this interval (5.7 × 7.5 cm in 2022 vs 5.6 × 7.5 cm in 2025). Ligamentous laxity, frequently invoked in the geriatric cases that dominate the prior literature, is a less plausible primary explanation in a 38-year-old patient.

### Proposed mechanism

The proposed mechanism is therefore likely to be obesity-associated, with chronically raised intra-abdominal pressure plausibly contributing to the progressive movement of additional viscera through a structurally stable but capacious paraumbilical fascial defect. Sequential extrusion of small bowel and omentum was followed by transverse colon and the distal antrum/pylorus (already herniated on baseline imaging in 2022), and ultimately by proximal migration of the gastric body, duodenal bulb and proximal D2 into the sac by 2025. Mechanical angulation of the distal stomach as it crossed the fascial defect produced extrinsic compression of the gastric outlet and the clinical syndrome of GOO.

### Role of cross-sectional imaging

Multi-detector contrast-enhanced CT is the preferred imaging modality in any patient with a giant or complex ventral hernia, and should be strongly considered in any patient with a ventral hernia accompanied by atypical upper-gastrointestinal symptoms (1, 18). CT defines the fascial defect in transverse and cranio-caudal dimensions, enumerates the herniated contents, identifies transition points along the gastrointestinal tract, characterises the abdominal-wall musculature, and enables formal volumetric assessment of loss of domain by the Tanaka (11) or Sabbagh (12) methods. In our patient, CT was decisive: it confirmed the gastric component within the sac, localised the transition to the distal antrum and pylorus, and excluded malignancy.

In retrospect, formal volumetric LOD assessment by the Tanaka and Sabbagh methods (defined in the Case Presentation) would have been preferable to qualitative review, as these are now widely advocated as standard pre-operative metrics in complex ventral hernia (11, 12, 18). Qualitative review of the CT in our patient suggested that hernia sac volume was below both thresholds, and intra-operative findings supported this; primary fascial closure with retromuscular mesh augmentation was achieved without tension and without recourse to chemical component separation, botulinum-toxin chemodenervation or progressive pneumoperitoneum (19, 20). The absence of formal volumetric LOD calculation is nevertheless a limitation of this report.

### Biochemistry

Hypokalaemia (potassium 2.78 mmol/L on admission) and mild hypoalbuminaemia (34 g/L) were the only biochemical abnormalities, despite a year of recurrent vomiting.

### Pre-operative nutritional optimisation

Although the patient's pre-operative weight (89 kg) represented a 30% reduction from his peak, formal nutritional assessment using serial BIA and hand-grip dynamometry suggested that lean body mass and functional muscle strength were preserved and improving on NJ feeding (serial values detailed in the Case Presentation). These findings argued against clinically significant sarcopenia and supported proceeding with definitive repair after a short prehabilitation interval rather than deferring further.

### Operative strategy: retromuscular (rives-stoppa) repair

The repair of giant ventral hernias is a recognised technical challenge, particularly when the defect is large enough to raise concern for loss of domain (18–20). The European Hernia Society defines a giant ventral hernia as a defect >10 cm in width regardless of LOD (1). In this patient, the intra-operative defect was 5 × 10 cm (transverse  ×  cranio-caudal) with healthy contents that reduced without tension, and pre-operative imaging did not suggest true LOD; consequently, adjuncts such as pre-operative botulinum-toxin chemodenervation, progressive pneumoperitoneum and posterior component separation were not required (18–20). When such adjuncts are required, contemporary evidence supports their integration into a staged or combined strategy with retromuscular mesh placement to maximise tension-free fascial closure (18–20).

The retromuscular sublay (Rives–Stoppa) mesh repair was selected as the procedure of choice. The Rives–Stoppa technique is widely regarded as the contemporary reference standard for open midline ventral hernia repair: it provides a wide, well-vascularised retromuscular plane for mesh placement, isolation of mesh from the peritoneal cavity, and durable mechanical reinforcement of the linea alba, and is associated with lower recurrence rates compared with onlay or pure suture repair (1, 19–21). A macroporous polypropylene-based mesh with at least 3 cm of fascial overlap on all sides was used, and the linea alba was reapproximated with looped polyamide 1 to re-establish a functional midline. The 3 cm minimum reflects the overlap originally described in Stoppa-type retromuscular repairs and remains within the range of acceptable practice in the European and American Hernia Society guidelines (1); however, more recent expert consensus and systematic reviews increasingly favour wider overlap of at least 5 cm where anatomically achievable, based on reduced recurrence in larger defects (19, 20). In our patient, the available retromuscular plane was limited by focal attenuation of the posterior rectus sheath at the apex of the defect, which constrained the size of mesh that could be safely deployed without breaching the posterior layer; this trade-off is described in detail in the operative narrative. The rationale for selecting a composite mesh in the retrorectus plane is detailed in the operative description and reflects the focal attenuation of the posterior rectus sheath in our patient.

### Perioperative cardiorespiratory considerations

Elective post-operative ventilation for 24 h in the intensive care unit was considered prudent given the size of the original sac, the volume of reduced viscera, the patient's peak class III obesity and severe obstructive sleep apnoea, and the recognised risk of intra-abdominal hypertension and post-operative respiratory failure after repair of giant ventral hernias (1, 14, 20). Early ambulation, enhanced-recovery feeding and physiotherapy completed the post-operative pathway.

### Obesity, hernia recurrence and the role of bariatric surgery

Class III obesity is independently associated with an increased risk of primary and recurrent ventral hernia, larger defect size, surgical-site occurrence and recurrence after hernia repair (16, 17, 22). Contemporary practice guidelines therefore advocate weight reduction increasingly via metabolic and bariatric surgery prior to elective ventral hernia repair in stable patients (1, 22–24). In the setting of recurrent obstruction, hernia incarceration or compromise of the abdominal wall, however, surgical urgency necessarily takes precedence (1, 22). The present case illustrates this principle: bariatric surgery had been deferred for several years because of active tobacco use and poor CPAP adherence, but the development of frank GOO mandated urgent hernia repair before any further weight optimisation could be considered.

Concomitant bariatric and ventral hernia repair has been described, and its risk–benefit profile is most favourable when the hernia is small or the bariatric procedure is laparoscopic; for giant ventral hernias such as the present case a staged approach is generally preferred (22–24). The patient has accordingly been re-referred for bariatric assessment once his abdominal wall has consolidated, reinforcing the bidirectional relationship between obesity management and complex hernia care.

The clinical course following hernia repair underscored this point: with mechanical obstruction relieved and oral intake unrestricted, the patient regained 29 kg over approximately five months (from 89 kg to 118 kg). This rapid post-operative weight regain reflects both recovery of caloric intake from a state of partial cachexia and the re-emergence of his underlying obesity, and is consistent with the recognised physiological tendency for body weight to return toward its pre-illness set-point once a metabolic stressor is removed. It also raises the prospect of recurrence of the hernia in the medium term and reinforces the urgency of progression to bariatric surgery once wound consolidation permits.

### Lessons learned and changes to our management approach

This case has prompted us to formalise three changes to our outpatient pathway for patients with concomitant severe obesity and complex ventral hernia. First, we monitor these patients more proactively, with a lower threshold for repeat cross-sectional imaging and earlier multidisciplinary review when any sudden or progressive unintentional weight loss is reported; in retrospect, such weight loss may paradoxically signal the onset of mechanical obstruction within the hernial sac rather than reflect successful lifestyle modification. Second, structured obesity management has now been integrated into every stage of the hernia pathway rather than treated as a prerequisite to be completed before surgical referral; in the present patient, post-operative weight management is coordinated jointly with dietetics and physiotherapy (including supervised resistance training to protect lean mass), with concurrent consideration of GLP-1/GIP receptor agonist therapy (tirzepatide) as a pharmacological adjunct while definitive bariatric surgical assessment is reorganised. Third, this experience has reinforced the value of explicit shared decision-making and documentation when bariatric surgery is recommended but declined, so that an active follow-up plan with defined imaging and clinical review intervals is articulated rather than a passive ‘await symptoms’ default. We acknowledge the broader observation that access to both medical and surgical weight-loss therapies remains constrained by regulatory and institutional factors in many jurisdictions; while these systemic issues lie beyond the scope of an individual case report, we believe the changes outlined above can be implemented within most hernia clinics caring for patients with severe obesity.

### Limitations

This is a single case and therefore cannot establish causality or generalisability. Reported defect dimensions varied between clinic, imaging and intra-operative measurements, reflecting the inherent difficulty of sizing a giant hernia in an obese abdomen. Formal volumetric loss-of-domain assessment (Tanaka or Sabbagh) was not performed pre-operatively, and intra-abdominal pressure was monitored indirectly via airway pressure, urine output and clinical signs rather than by intravesical manometry. The most recent clinical review was on 4 May 2026, providing approximately five months of post-operative follow-up. While the patient was asymptomatic with no clinical evidence of recurrence at this time-point, recurrence after open Rives-Stoppa repair of giant ventral hernias typically declares itself over years rather than months particularly in the setting of the marked post-operative weight regain documented here and longer-term follow-up beyond one year, ideally to five years, will be required before any definitive statement on durability can be made. Nonetheless, the case offers a clear illustration of an under-recognised mechanism of GOO in a young obese patient and supports an existing call in the literature for cross-sectional imaging in any patient with a giant ventral hernia and upper gastrointestinal symptoms. Several further limitations of our peri-operative monitoring should be acknowledged explicitly. Bioelectrical impedance analysis on the InBody platform is reproducible and widely available but is not the gold standard for body-composition assessment; dual-energy x-ray absorptiometry (DEXA), CT-based skeletal-muscle-area indexing at the L3 level or magnetic resonance imaging provide more accurate quantification of skeletal muscle mass and were not used here. BIA-derived estimates are also influenced by hydration status, which is likely to have been variable during the period of nasojejunal enteral feeding and fluid resuscitation; serial BIA trends in this setting should therefore be interpreted with caution. Hand-grip dynamometry, although reproducible and functionally informative, reflects forearm muscle strength and is an imperfect surrogate for whole-body sarcopenia. As already noted, intra-abdominal pressure was monitored indirectly rather than by intravesical manometry, and formal volumetric loss-of-domain assessment was not performed pre-operatively. Finally, while the patient is clinically well at the time of most recent review, the post-operative interval reported here remains short, and any inference about the durability of repair, the trajectory of weight regain or the eventual risk of GOO recurrence must be regarded as provisional and confined to this individual patient.

## Conclusion

GOO secondary to a stomach-containing paraumbilical hernia is a rare but clinically important entity that can occur in young patients with severe obesity through chronically raised intra-abdominal pressure and progressive defect enlargement, rather than solely in elderly patients with ligamentous laxity, as has previously dominated the literature. The combination of insidious post-prandial vomiting, learned self-induced emesis, profound unintentional weight loss and a giant irreducible paraumbilical hernia should prompt immediate multi-detector contrast-enhanced CT directed at the gastric outlet, with formal volumetric LOD assessment by the Tanaka or Sabbagh method. Objective pre-operative nutritional optimisation using serial BIA and grip-strength dynamometry, multidisciplinary preparation, an open Rives-Stoppa retrorectus mesh repair and elective post-operative ventilation provided satisfactory short-term resolution, with longer-term follow-up required before durability can be confirmed in this patient. Future bariatric intervention is anticipated to address the underlying driver and to reduce the risk of recurrence.

## Patient perspective

The patient reported a substantial improvement in quality of life following surgery, with resolution of post-prandial vomiting, restoration of normal bowel function and the ability to eat without inducing emesis. He has consented to the publication of his anonymised case to assist the recognition of this entity in other obese patients with long-standing ventral hernias.

## Data Availability

The original contributions presented in the study are included in the article/Supplementary Material, further inquiries can be directed to the corresponding author.

## References

[1] Henriksen NA Montgomery A Kaufmann R Berrevoet F East B Fischer J. Guidelines for treatment of umbilical and epigastric hernias from the European Hernia Society and Americas Hernia Society. Br J Surg. (2020) 107(3):171–90. 10.1002/bjs.11489 31916607

[2] Kulaçoğlu H. Current options in umbilical hernia repair in adult patients. Ulus Cerrahi Derg. (2015) 31(3):157–61. 10.5152/UCD.2015.2955 26504420 PMC4605112

[3] Favela JG Argo MB McAllister J Waldrop CL Huerta S. Gastric outlet obstruction from stomach-containing groin hernias: case report and a systematic review. J Clin Med. (2024) 13(1):155. 10.3390/jcm13010155 PMC10779582 38202162

[4] Koop AH Palmer WC Stancampiano FF. Gastric outlet obstruction: a red flag, potentially manageable. Cleve Clin J Med. (2019) 86(5):345–53. 10.3949/ccjm.86a.18035 31066665

[5] Tringali A Giannetti A Adler DG. Endoscopic management of gastric outlet obstruction disease. Ann Gastroenterol. (2019) 32(4):330–7. 10.20524/aog.2019.0390 31263354 PMC6595925

[6] Mwangi PM Chalya PL Mabula JB Massinde A Jaka H. Gastric outlet obstruction at bugando medical centre in northwestern Tanzania: a prospective review of 184 cases. BMC Res Notes. (2013) 6:298. 10.1186/1756-0500-6-298 24067148 PMC3849005

[7] Lim K Hattori R. Gastric outlet obstruction secondary to paraumbilical hernia. Surgery. (2022) 171(2):e1–2. 10.1016/j.surg.2021.07.040 34404543

[8] Boyapati S Reichstein AC McLaurin S Tonkin A. Gastric outlet obstruction secondary to an incarcerated pylorus in an umbilical port site hernia. ACS Case Reviews in Surgery. (2022) 4(4):5–9. https://www.facs.org/media/ss5lftlw/02-boyapati-gastric-outlet-obstruction.pdf

[9] Salemis NS Nisotakis K Gourgiotis S Tsohataridis E. Gastric outlet obstruction with elevated serum pancreatic lipase secondary to an infraumbilical hernia. ANZ J Surg. (2014) 84(7-8):573–4. 10.1111/ans.12153

[10] Gagnier JJ Kienle G Altman DG Moher D Sox H Riley D. The CARE guidelines: consensus-based clinical case reporting guideline development. BMJ Case Rep. (2013);2013:bcr2013201554. 10.1136/bcr-2013-201554 PMC3844611 24228906

[11] Tanaka EY Yoo JH Rodrigues AJ Jr Utiyama EM Birolini D Rasslan S. A computerized tomography scan method for calculating the hernia sac and abdominal cavity volume in complex large incisional hernia with loss of domain. Hernia. 2010;14(1):63–9. 10.1007/s10029-009-0560-8 19756913

[12] Sabbagh C Dumont F Robert B Badaoui R Verhaeghe P Regimbeau JM. Peritoneal volume is predictive of tension-free fascia closure of large incisional hernias with loss of domain: a prospective study. Hernia. (2011) 15(5):559–65. 10.1007/s10029-011-0832-y 21584816

[13] Clark ST Malietzis G Grove TN Jenkins JT Windsor ACJ Kontovounisios C. The emerging role of sarcopenia as a prognostic indicator in patients undergoing abdominal wall hernia repairs: a systematic review of the literature. Hernia. (2020) 24(6):1361–70. 10.1007/s10029-020-02179-6 32300901 PMC7701077

[14] Kirkpatrick AW Roberts DJ De Waele J Jaeschke R Malbrain MLNG De Keulenaer B. Intra-abdominal hypertension and the abdominal compartment syndrome: updated consensus definitions and clinical practice guidelines from the world society of the abdominal compartment syndrome. Intensive Care Med. (2013) 39(7):1190–206. 10.1007/s00134-013-2906-z 23673399 PMC3680657

[15] Costantini B Lecointre L Loroch M Bellier B Mutter D Marescaux J. Parastomal hernia with gastric outlet obstruction: a case report and literature review. Int J Surg Case Rep. (2018) 49:96–9. 10.1016/j.ijscr.2018.06.013 30046442 PMC6054266

[16] Sauerland S Korenkov M Kleinen T Arndt M Paul A. Obesity is a risk factor for recurrence after incisional hernia repair. Hernia. (2004) 8(1):42–6. 10.1007/s10029-003-0161-x 13680307

[17] Köckerling F Hoffmann H Adolf D Reinpold W Kirchhoff P Mayer F. Female sex, BMI > 35 and shorter time to recurrence are independent risk factors for re-recurrence of incisional hernia after open ventral hernia repair. Hernia. (2018) 22(6):1077–86. 10.1007/s10029-018-1818-930155571

[18] Parker SG Halligan S Liang MK Muysoms FE Adrales GL Boutall A. Definitions for loss of domain: an international delphi consensus of expert surgeons. World J Surg. (2020) 44(4):1070–8. 10.1007/s00268-019-05317-z 31848677

[19] Liang MK Holihan JL Itani K Alawadi ZM Gonzalez JR Askenasy EP. Ventral hernia management: expert consensus guided by systematic review. Ann Surg. (2017) 265(1):80–9. 10.1097/SLA.0000000000001701 28009730

[20] Bittner R Bansal VK Bingener J Bisgaard T Buhck H Dudai M. Update of guidelines for laparoscopic treatment of ventral and incisional abdominal wall hernias [international endohernia society (IEHS)] – part B. Surg Endosc. (2019) 33(11):3511–49. 10.1007/s00464-019-06908-6 31292742 PMC6795640

[21] Holihan JL Bondre I Askenasy EP Greenberg JA Keith JN Martindale RG. Sublay versus underlay in open ventral hernia repair. J Surg Res. (2015) 199(1):141–6. 10.1016/j.jss.2015.04.015 27083944

[22] Borbély Y Zerkowski J Altmeier J Eschenburg A Kröll D Nett P. Complex hernias in patients with morbid obesity. Hernia. (2017) 21(5):743–9. 10.1007/s10029-017-1645-4 28302520

[23] Sneiders D Yurtkap Y Kroese LF Kleinrensink GJ Lange JF Gillion JF. Hernia repair in the bariatric patient: a systematic review and meta-analysis. Surg Obes Relat Dis. (2024) 20(2):205–15. 10.1016/j.soard.2023.10.005 37973424

[24] Restrepo-Rodas G Barajas-Gamboa JS Corcelles R. Management of abdominal wall hernias in bariatric patients: a narrative review. Ann Laparosc Endosc Surg. (2024) 9:17. 10.21037/ales-24-12

